# Draft Genome Sequence of *Tetragenococcus halophilus* Strain FBL3, a Probiotic Bacterium Isolated from Galchijeot, a Salted Fermented Food, in the Republic of Korea

**DOI:** 10.1128/genomeA.00304-17

**Published:** 2017-05-04

**Authors:** Eiseul Kim, Jae-Hwan Kim, Seung-Min Yang, Seung-Man Suh, Hyun-Joong Kim, Chang-Gyeom Kim, Dong-Won Choo, Hae-Yeong Kim

**Affiliations:** a Institute of Life Sciences and Resources Graduate School of Biotechnology, Kyung Hee University, Yongin, Republic of Korea; b Department of Bioinformatics and Biosystems, Korea Polytechnics, Seongnam, Republic of Korea

## Abstract

*Tetragenococcus halophilus* strain FBL3 is a lactic acid bacterium isolated from galchijeot, a fermented food made from the salted guts of the hairtail fish, in the Republic of Korea. The draft genome of *T. halophilus* strain FBL3 comprised 87 contigs (≥1 kb) with a total size of 2,420,904 bp and a G+C content of 38.5%.

## GENOME ANNOUNCEMENT

*Tetragenococcus halophilus* (previously classified as *Pediococcus halophilus*) is a catalase- and oxidase-negative, Gram-positive, nonmotile, halophilic lactic acid bacterium with spherical cells (0.5 to 0.8 µm) (1). *Tetragenococcus* spp. are frequently isolated from salt-rich environments, such as fermented foods and human ear secretions, as well as sugar-rich environments (2, 3). The entire genome of *T. halophilus* strain NBRC 12172 (AP012046.1) consisted of a circular chromosome of 2,562,720 bp.

*T. halophilus* strain FBL3 was isolated from galchijeot, a fermented food made from the salted guts of the hairtail fish, in the Republic of Korea, and the 16S rRNA gene sequence of the strain showed 100% identity with the partial sequence of *T. halophilus* subsp. *halophilus* strain JCM 5888 (LC071840.1).

Genomic DNA was extracted with the G-spin genomic extraction kit (Intron Biotechnology Co., Republic of Korea) and its quality was evaluated using an Agilent 2100 bioanalyzer with the high-sensitivity DNA kit. Draft genome sequencing was performed using an Ion Torrent Personal Genome Machine (Life Technologies, Inc., Germany) and a 318 semiconductor chip with 400-bp sequencing reads (4). A total of 4,110,289 reads, with an average read length of 310 bp, were obtained. Reference-based assembly was carried out using SPAdes version 3.1.0 with *T. halophilus* NBRC 12172 (AP012046.1) as the reference genome. Assembly of the reads resulted in 87 contigs ≥1 kb (1 to 144,210 kb). The total draft genome size was 2,420,904 bp with a G+C content of 38.5%.

The genome sequence was annotated with the Rapid Annotations using Subsystems Technology (RAST) server (5, 6). The draft genome has 2,378 coding sequences, 58 RNAs, and 323 subsystems. Genes involved in acid tolerance (F1F0-ATPase system), oxidative stress resistance (thioredoxin, glutathione reductase, NADH-peroxidase, RecA protein), osmotic stress response (osmoregulation, ectoine biosynthesis and regulation, choline and betaine biosynthesis), osmotolerance (GTP pyrophosphokinase), adhesion and colicin V production, as well as genes encoding resistance to cobalt-zinc-cadmium, fluoroquinolones, arsenic, and cadmium, were present in the genome.

The following were also detected in the *T. halophilus* strain FBL3 genome: ethanolamine utilization proteins, amino acid transfer proteins, and various phosphotransferase system proteins (*N*-acetylgalactosamine, galactosamine, trehalose, and mannitol). Compared to the *T. halophilus* NBRC 12172 genome, approximately 60 genes are missing.

### Accession number(s).

This whole-genome shotgun project has been deposited in DDBJ/ENA/GenBank under the accession number LSFG00000000. The version described in this paper is the first version, LSFG01000000.
